# COGNITIVE AGING AMONG OLDER LATINOS IN THE US, MEXICO, AND BRAZIL

**DOI:** 10.1093/geroni/igae098.1151

**Published:** 2024-12-31

**Authors:** Jacqueline Angel, Flávia Andrade, Jacqueline Angel

**Affiliations:** Chair; Co-Chair; Discussant

## Abstract

Amidst rapid demographic transitions in the Americas, where dementia prevalence is poised to double by 2050, this symposium offers a crucial exploration of cognitive health in the Americas. Leveraging data from Brazil, Mexico, and the US, it emphasizes understanding measurement, contextual factors, diversity of experiences, and associated costs in addressing this pressing global challenge. Cantu et al. scrutinize how gender influences cognitive assessments in older adults in the US and Mexico, unveiling potential biases in reporting and underscoring the need for accurate diagnosis. Exploring cultural resources among very old Mexican American immigrants, Kim and Silverstein shed light on their impact on psychological and cognitive well-being, highlighting the role of cultural context in shaping health outcomes and offering insights into protective factors against cognitive decline. Estimating the economic impact of dementia, Mudrazija et al. assess lost wages, medical care spending, and unpaid care costs for African American and Latino adults in the US and their caregivers, projecting these costs through 2060. García-Chanes and Lopez-Ortega characterize the sample of older adults in Mexico living with dementia in 2016 and the specific sources of social and economic support they receive. Lastly, Chen et al. examine the association between Internet use and cognitive health among Brazilian older adults, suggesting the potential of technology in promoting cognitive well-being and stressing the importance of innovative approaches in enhancing cognitive health outcomes. By synthesizing diverse perspectives and data sources, this symposium offers invaluable insights for developing targeted interventions and policies to address the challenges posed by dementia.

